# Thermoelectric Performance of Mechanically Mixed Bi_x_Sb_2-x_Te_3_—ABS Composites

**DOI:** 10.3390/ma14071706

**Published:** 2021-03-30

**Authors:** Zacharias Viskadourakis, Argiri Drymiskianaki, Vassilis M. Papadakis, Ioanna Ioannou, Theodora Kyratsi, George Kenanakis

**Affiliations:** 1 Institute of Electronic Structure and Laser (IESL)—Foundation for Research and Technology—Hellas (FORTH), 100 N. Plastira, Vassilika Vouton, GR-70013 Heraklion, Crete, Greece; billyp@iesl.forth.gr; 2 Physics Department, University of Crete, Vassilika Vouton, GR-70013 Heraklion, Crete, Greece; ph4055@edu.physics.uoc.gr; 3 Department of Mechanical & Manufacturing Engineering, University of Cyprus, 75 Kallipoleos Ave., P.O. Box 20537, Nicosia 1678, Cyprus; gianna1992@live.com (I.I.); kyratsi@ucy.ac.cy (T.K.)

**Keywords:** thermoelectric materials, Bismuth Antimony Telluride, Seebeck coefficient, polymer-thermoelectric material blends, polymeric nanocomposites

## Abstract

In the current study, polymer-based composites, consisting of Acrylonitrile Butadiene Styrene (ABS) and Bismuth Antimony Telluride (Bi_x_Sb_2−x_Te_3_), were produced using mechanical mixing and hot pressing. These composites were investigated regarding their electrical resistivity and Seebeck coefficient, with respect to Bi doping and Bi_x_Sb_2-x_Te_3_ loading into the composite. Experimental results showed that their thermoelectric performance is comparable—or even superior, in some cases—to reported thermoelectric polymer composites that have been produced using other complex techniques. Consequently, mechanically mixed polymer-based thermoelectric materials could be an efficient method for low-cost and large-scale production of polymer composites for potential thermoelectric applications.

## 1. Introduction

In the last decade, thermoelectric (TE) materials have gained considerable attention in waste heat recovery applications due to their capability to convert heat to electricity utilizing the Seebeck effect. The performance of a TE material can be determined through the thermoelectric figure of merit Z − $ZT=S^{2}T/\kappa \rho$—where S is the Seebeck coefficient, κ is thermal conductivity, ρ is electrical resistivity, and T is the absolute temperature. Moreover, thermal conductivity consists of two parts: an electronic one and another one coming from the lattice (phononic), so that $\kappa =\;\kappa_{el}+\;k_{l}$, where $\kappa_{el}$ is the electronic component and $\kappa_{l}$ is the lattice component. Thus, an efficient thermoelectric material appropriate for commercial applications (ZT>1) must exhibit high S, along with low κ and ρ. However, all three physical parameters are interrelated such that materials with high Seebeck coefficient values usually also exhibit high resistivity, and materials with low resistivity show a low Seebeck coefficient, leading to reduced ZT values. On the other hand, low resistivity materials usually exhibit a high charge carrier concentration, which results in increased thermal conductivity because high carrier concentration increases the electronic thermal conductivity. Due to this diverging interconnection among S, ρ, and κ, the realization of high ZT materials becomes challenging, since fine optimization between those three quantities is required [1].

Today, state-of-the-art thermoelectrics are mostly inorganic materials, such as tellurides, oxides, skutterudites, half-Heusler alloys, silicon–germanium composites, etc. [2,3,4]. All these materials exhibit ZT>1 at a specific temperature regime, and they are all used in commercially available thermoelectric power generators. However, these high efficiency inorganic thermoelectrics exhibit serious disadvantages, such as toxicity, high production cost, complicated production process, etc. Moreover, these materials are not mechanically flexible so commercially available TE generators cannot be optimally attached to arbitrary-shaped heating sources, further limiting their heat-to-power conversion capability.

In this context, TE polymers could be an effective alternative choice for low-cost and flexible TE devices. Whether they are intrinsic or doped, such polymers exhibit very effective thermoelectric performance, especially near room temperature [5,6,7]. In particular, such TE polymers show increased electrical conductivity; however, they also exhibit low thermal conductivity. Due to their construction of mainly long chains of carbons, polymers sustain very low, phononic dominated, thermal conductivity, which is hardly affected by charge carrier concentration. Therefore, TE polymers provide good chances for decoupling between resistivity and thermal conductivity towards the enhancement of their thermoelectric performance. Furthermore, combined with their efficient mechanical properties, such materials become promising candidates for flexible TE applications. However, challenges regarding the massive production of those polymers impeded their use in commercial and large-scale applications [8,9].

An alternative method proposed is to mix a thermoelectric material with a polymer. In general, polymer–TE composites can be prepared in three different ways: in situ mixing, solution mixing, and mechanical mixing [10]. Such mixing results in a final composite with appropriate TE performance, due to the TE inclusions and the mechanical properties of the polymer. In this context, several attempts have been reported in the literature that resulted in polymer composites with exceptional TE performance [11,12,13,14]. In situ mixing includes several chemical procedures and steps [15]. On the other hand, the processes employed in solution mixing include using solvents and resins to dissolve polymers and inorganic fillers [16], liquid dispersers and ultrasonic sonicators for uniform distribution of the TE material into the composite, and polymer plasticizers to improve the encapsulation of the TE material into the polymer matrix etc. Such procedures are very effective in lab-scale production; however, they most likely are not applicable for large scale production. In contrast, mechanical mixing is a simple process in which nothing else is required, apart from the mixing materials in powder form.

Considering all of the above, we employed mechanical mixing and hot-pressing to produce a polymer-based composite, which can be potentially used for TE applications. In particular, we mixed a widely used polymer, Acrylonitrile Butadiene Styrene (ABS), and a state-of-the art TE material, Bismuth Antimony Telluride Bi_x_Sb_2-x_Te_3_, using a simple mixing homogenizer. After mixing, the resulting mixture was hot-pressed so that a thin foil was produced. Both mixing and pressing procedures are simple, and large quantities of mixtures can be produced in relatively short time. Moreover, they are already used on a factory scale by the polymer industry. Several foils were produced, with various Bi doping levels, as well as different amounts of TE material and ABS. All foils were investigated regarding their thermoelectric performance. It was found that all produced foils exhibited thermoelectric behavior comparable, or even superior, to the thermoelectric performance of other polymer-based thermoelectric nanocomposites, which are produced through more complicated approaches. Therefore, herein it is shown that polymer-based TE composites with effective TE performance can be massively produced, in a short time and for a low cost.

## 2. Experimental Details

**Figure 1:**
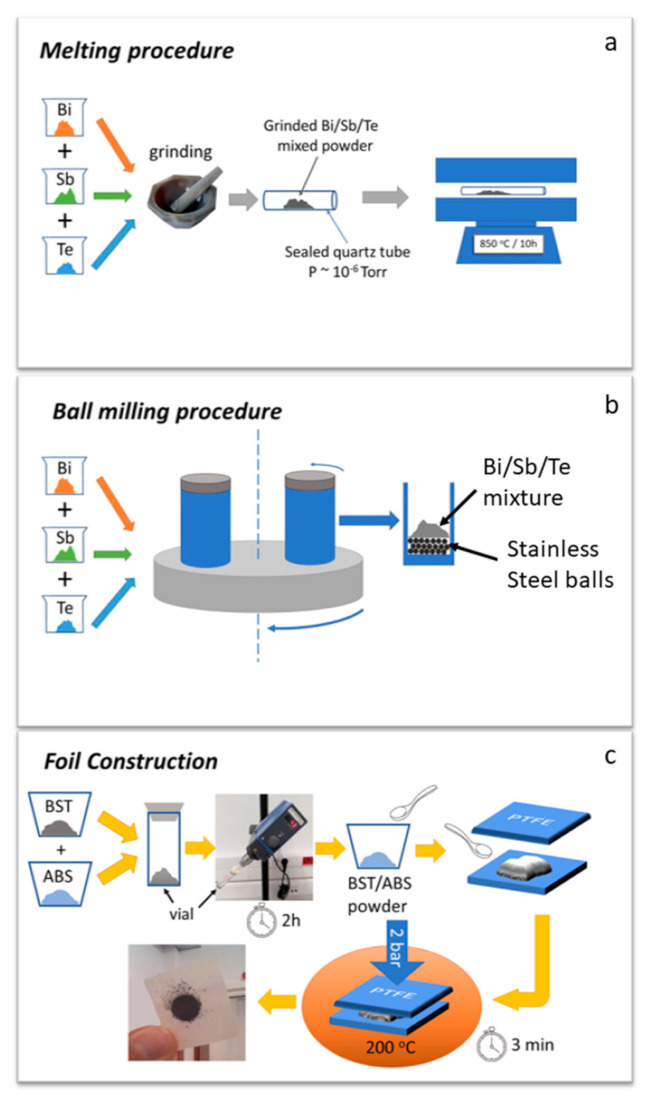
Schematic representation of Bi_x_Sb_2-x_Te_3_ (BST) materials preparation. (**a**) Melting procedure. (**b**) Mechanical alloying procedure. (**c**) Schematic representation of BST foil construction. The constructed foil is presented in the final step (it is glued on weighing paper for photography purposes).

High purity Bi_x_Sb_2-x_Te_3_ (BST) powders $\left(0.2\leq x\;\leq 0.5\right)$ were produced by two different methods: melting and mechanical alloying [17,18,19]. The p-type BST x = 0.3 powder was fabricated by melting, while BST (x = 0.2, 0.4, 0.5) solid solutions were prepared by the mechanical alloying method. For the melting synthesis process (Figure 1a)**,** appropriate amounts of high purity (5 N) Bi, Sb, and Te powders mixed together in a mortar. Then, the mixture was put into a quartz tube. The tube was evacuated and sealed before it was put into a furnace at 800 °C for 10 h. For the mechanical alloying process (Figure 1b), appropriate amounts of the Bi, Sb, and Te powders were loaded into a cup of a planetary ball milling machine (Pulviresette 6, Fritsch GmbH, Berlin, Germany), along with stainless steel balls (10 mm in diameter). The ball milling process took place in an oxygen-free environment to avoid oxidation of the starting materials. The milling speed was set at 300 rpm, the milling time was set at 20 h, and the ball to material ratio was 10:1. The produced powders were used as starting ingredients to make mixtures, in combination with industrial grade ABS fine powder provided by INEOS Styrolution (Frankfurt, Germany), under the name Terluran Hi-10. Each mixture consisted of appropriate amounts of BST and ABS, for a BST/ABS mass ratio in the range of $75/25\;\leq \;m_{BST}/m_{ABS}\leq 90/10$.

A very simple method was followed for the formation and homogenization of the above-mentioned blends (Figure 1c). After being properly weighed, ABS and BST powders were put into a glass vial, attached to a mechanically rotating stick (rotation speed was ~50 rpm, rotation time ~2 h), and tilted by ~45° with respect to the vertical axis. Such a set-up closely resembles the rotation of a cement mixer. Rotation speed was kept as low as possible in order to prevent heating of the blend due to high rotation speeds. Tilted rotation of the vial resulted in homogenization of the mixed powders, without using stirrers, solvents, or ultrasonic homogenizers.

After that, a trace of the produced homogenized blended powder was placed between two pieces of Polytetrafluoroethylene (PTFE) foil. This sandwich-like combination was placed in a hot press and a pressure of 2 bar was applied, at 200 °C, for 3 min. PTFE is selected on purpose, since its melting point is ~300 °C; thus, it is well above the temperature used in the current procedure. The combination of pressure and heat melts the ABS resulting in the formation of a thin, foil-like sample, with an average thickness of ~200 µm. The produced foils were circular, with typical diameter of ~20 mm. From each foil, two parallelepiped pieces were extracted: one for resistivity measurements with typical dimensions of 5 mm × 3 mm × 0.2 mm and another for Seeebeck coefficient measurements with dimensions of 10 mm × 4 mm × 0.2 mm.

The crystal structure, of all produced foils, was characterized by the X-ray diffraction (XRD) technique, using a Rigaku (RINT 2000; Rigaku, Tokyo, Japan) Cu *Kα* (λ = 1.5418 Å) X-ray diffractometer, while their surface morphology was studied by means of a field emission scanning electron microscope—SEM (FE-SEM, JEOL JSM-7000; JEOL Ltd., Tokyo, Japan), with Energy Dispersive Spectrometer (EDS) equipped.

Furthermore, Raman experiments were performed at room temperature using a Horiba LabRAM HR Evolution confocal micro-spectrometer (HORIBA FRANCE SAS, Longjumeau, France) equipped with a 532 nm laser that can provide ~14 mW power on the sample surface.

Electrical resistivity measurements were performed, employing the conventional 4-wire technique (Figure 5c). For the purposes of the measurement, Cu wires were attached to the surface of the sample using conductive silver paste. In order to suppress any contact effects, the samples were subjected to heat treatment at 150 °C for 20 min, and ohmic contacts were formatted between silver paste and samples. The ohmic character of the contacts was confirmed through corresponding I-V measurements. Moreover, electrical resistivity was measured against temperature, from room temperature up to ~130 °C, using a tube furnace and a home-built apparatus.

The Seebeck coefficient was also measured with respect to the temperature, employing the well-known steady state technique (Figure 6c). One side of the sample was attached to a surface with constant temperature, while a micro-heating element was attached on the other side. As DC voltage was applied to the micro-heater, it heated the sample from one side to the other, resulting in the establishment of a temperature difference ΔT, along the sample. This difference was measured using two type-E thermocouples. At the same time, due to the presence of such ΔT, a voltage drop ΔV occurred, which was measured using a sensitive voltmeter. The Seebeck coefficient was determined through the relation S= Sref−ΔVΔT, where $S_{ref}$ is the absolute Seebeck coefficient of the leads, which are used to measure the ΔV. Details regarding the technique can be found elsewhere [20].

Finally, it is worth saying that mechanical properties of the prepared foil-like composites are under investigation and will be presented in future work.

## 3. Results and Discussion

**Figure 2:**
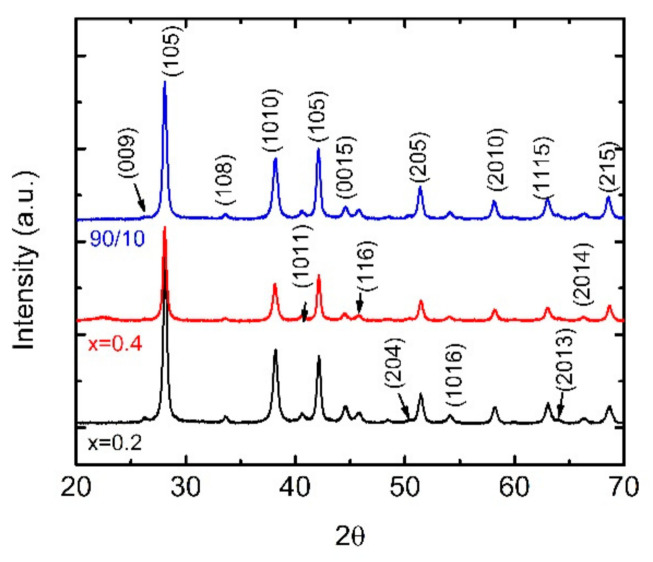
X-ray diffraction (XRD) patterns for x = 0.2 (black line) and x = 0.4 (red line) BST/Acrylonitrile Butadiene Styrene (ABS) blends mBSTmABS= 8020. Blue line corresponds to BST (x = 0.4)/ABS blend with mBSTmABS= 9010. Miller indices are similar for all spectra; however, they are presented for all three spectra, for clarity.

Figure 2 shows typical XRD patterns for BST/ABS blends. All diffraction peaks observed are indexed to the rhombohedral phase, Bi_x_Sb_2-x_Te_3_, consistent with the literature [17]. No secondary phases are observed within the resolution of the instrument. For the mechanical-alloying processed samples, the crystallite size was estimated using the Scherrer formula $d=\;\frac{0.9\;\lambda }{\beta \;cos\theta }\;$where *d*, *λ*, *θ*, and *β* are the crystallite size, the X-ray wavelength (1.5418 Å), the Bragg diffraction angle, and the full width of the half maximum (FWHM) of the diffraction peak, respectively. In our case, crystallite size was statistically determined from the width of the (105), (1010), (110), and (205) reflections of the BST phase at 2θ = 28.12°, 38.18°, 42.11°, and 44.57°, respectively and it was found to be ~20 nm. The estimated value corroborates with grain dimensions obtained for mechanically alloying Bi_2_Te_3_ samples previously reported [19]. Moreover, there is not any difference observed, neither with increasing doping nor with increasing BST loading.

**Figure 3:**
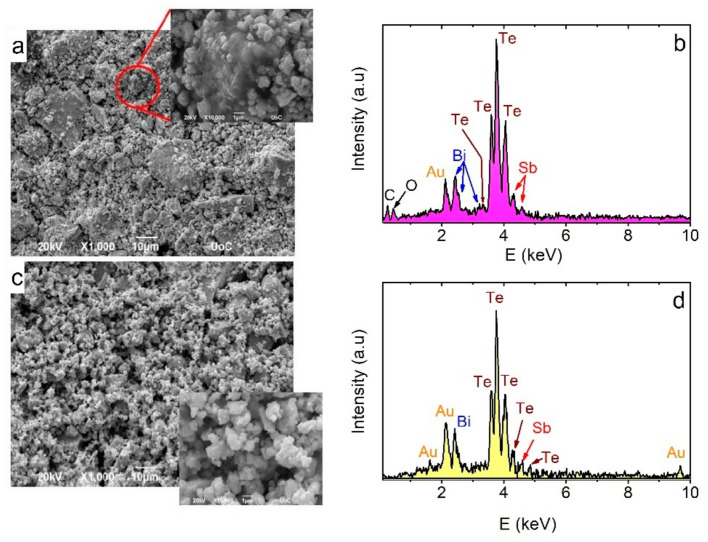
(**a**) Scanning electron microscopy (SEM) micrograph for BST (x = 0.4)/ABS foil mBSTmABS= 8020. The existence of ABS is observed in the inset. (**b**) Corresponding Energy Dispersive Spectrum (EDS), in which characteristic peaks of Bi, Te, Sb, C, O, and Au elements are observed. (**c**) SEM micrograph for pure BST (x = 0.4) powder. Details regarding the grain size and the porosity of the powder are observed in the inset. (**d**) Corresponding EDS spectrum of the BST (x = 0.4) powder. It is noted that Au peaks correspond to Au sputtered on the samples’ surface, in order to avoid charging effects during SEM experiments.

Figure 3a shows a typical SEM micrograph for BST/ABS x = 0.3 foil mBSTmABS= 8020. The foil exhibits a grainy surface consisting of large (a few µm) and small (hundreds of nm) grain clusters. The ABS component is also observed in some cases (inset, Figure 3a) in which the BST grains are encapsulated. Moreover, empty space exists among clusters, indicating the high porosity of the foil. Corresponding EDX spectrum is shown in Figure 3b. Peaks attributed to Bi, Sb, Te, C, O, and Au elements are shown, corroborating the existence of BST and ABS components in the composite. Similar SEM micrographs and EDS spectra were obtained for all BST/ABS foils and investigated, regardless of the Bi doping or the m_BST_/m_ABS_ ratio. On the other hand, corresponding pure BST powder (Figure 3b) also shows large porosity, while the average cluster grain size lays in the range of a few hundreds of nm (e.g., inset Figure 3b). Thus, the existence of large grain clusters in foils, could plausibly be attributed to the pressure applied along the foil production. Figure 3d shows the EDX spectrum of the BST powder, in which all Bi, Se, Te, and Au elements are presented. Apart from the absence of C and O peaks, this spectrum is identical to that obtained for the BST/ABS composite, indicating that BST powder is not affected by the hot-pressing procedure.

**Figure 4:**
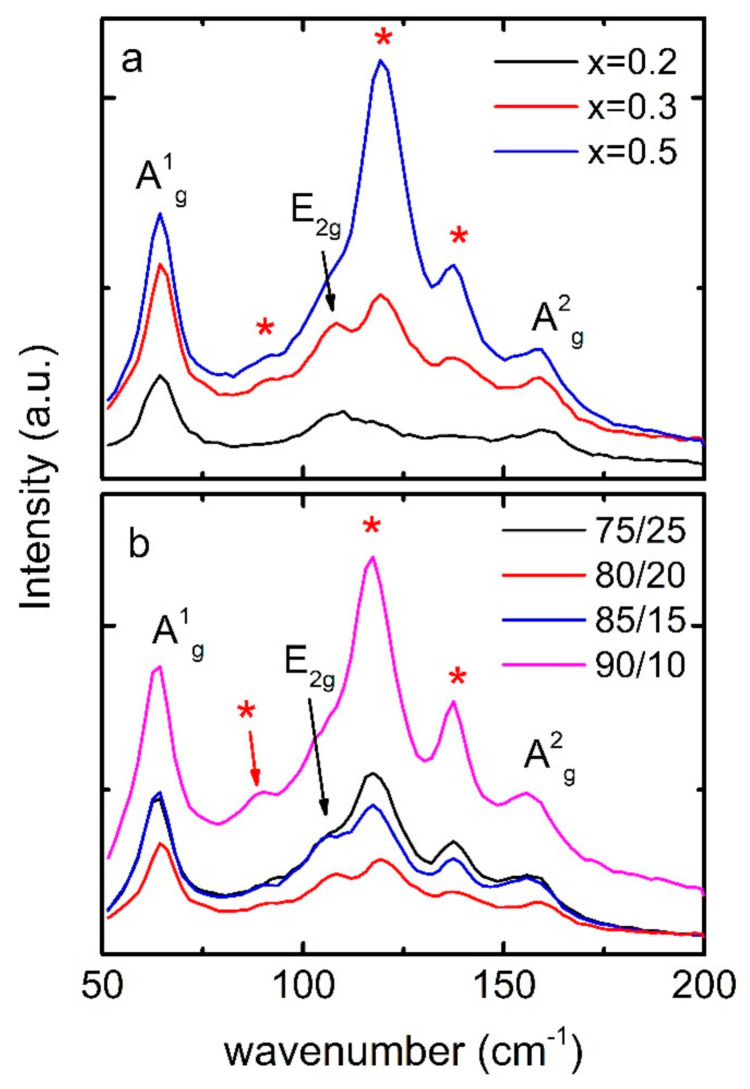
(**a**) Raman spectra BST/ABS blends mBSTmABS= 8020, with various Bi dopings. Peaks pointed with red stars correspond to vibration modes of Bi_2_Te_3_ structure. (**b**) Raman spectra for blends with different mass ratios (BST, x = 0.4).

Figure 4a depicts Raman spectra BST/ABS blends mBSTmABS= 8020, with various Bi dopings. For x = 0.2, three peaks are hardly seen at 64 cm^−1^, 107 cm^−1^ and 161 cm ^−1^, which correspond to the Sb/Bi–Te vibrational modes A^1^_g_, E^1^_g_, and A^2^_g_, respectively [21,22,23]. With increasing Bi doping these peaks are enhanced while other peaks show up at 89 cm^−1^, 120 cm^−1^, and 138 cm^−1^. These peaks match corresponding values, which are reported for Bi_2_Te_3_ powders [24,25,26]. Therefore, all peaks are identified as Bi_x_Sb_2−x_Te_3_ peaks. Figure 4b shows Raman spectra for blends with different mass ratios. All curves are similar to each other, despite the different intensity of the peaks.

**Figure 5:**
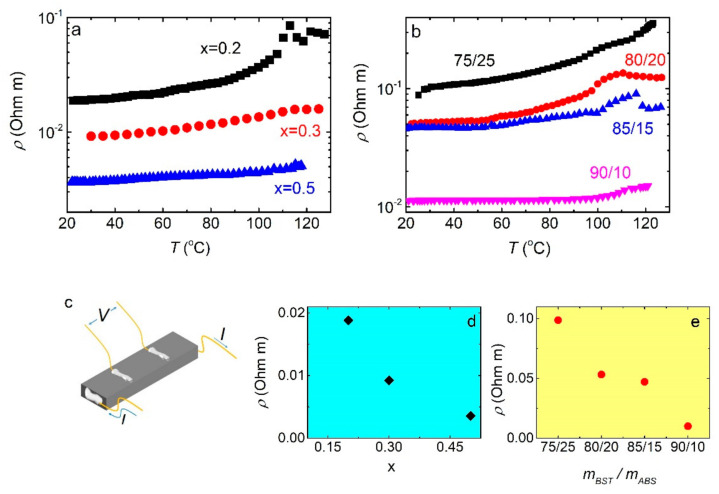
(**a**) Resistivity vs. temperature for BST/ABS blends mBSTmABS= 8020 for various Bi dopings. (**b**) Room temperature resistivity vs. Bi doping. (**c**) The wire configuration, for the resistivity measurement, is shown. (**d**) $\rho \;\left(T\right)$ curves for BST/ABS blends (BST x = 0.4) for different mass ratios. (**e**) Room temperature resistivity values vs. mass ratio, as extracted from panel **b**.

Figure 5a shows the resistivity $\rho \;\left(T\right)$ curves for BST/ABS blends mBSTmABS= 8020 with various Bi dopings. Resistivity increases with *T* in all cases, indicative of metallic behavior. Moreover, it is decreased with increasing Bi doping (Figure 5c). For the x = 0.2 blend, an anomaly is observed at ~110 °C, which is most likely associated with the glass transition of the ABS (~105 °C). Anomalies in the resistivity associated with the glass transition have also been reported for other polymer-based thermoelectric nanocomposites [20]. Absolute resistivity values are much lower than others reported for various polymer/BST composites [27,28,29]; nevertheless, it is 4–5 orders of magnitude larger than those reported for bulk BST samples [17], regardless the powder synthesis procedure followed. Such a huge difference could be attributed to the microstructure of the foils, as observed in SEM micrographs (Figure 3). BST powders used in this study consist of fine grains (mean grain size ~20 nm), which are not closely packed showing a sizable porosity. Both small grain size [30,31] and high porosity [32,33] could result in such resistivity enhancement. Finally, it is worth noting that the x = 0.5 blend exhibits low room temperature resistivity, comparable to that obtained for Polylactic Acid (PLA)/BST filament [34].

The effect of the BST/ABS mass ratio on the resistivity is pictured in Figure 5b (BST x = 0.4). All samples show metallic behavior, consistent with our previous observations. Moreover, resistivity decreases with increasing $m_{BST}/m_{ABS}$ ratio (Figure 5e). Minimum resistivity is observed for the maximum BST loading mBSTmABS= 9010. Absolute resistivity values are comparable to those reported for other BST/polymer blends [13,14,28,29,34,35]. Unfortunately, BST/ABS foils with higher $m_{BST}/m_{ABS}$ ratios cannot be acquired, since the produced foils are extremely fragile due to the very low ABS concentration. On the other hand, foils with lower BST loading exhibit rather high resistivity.

**Figure 6:**
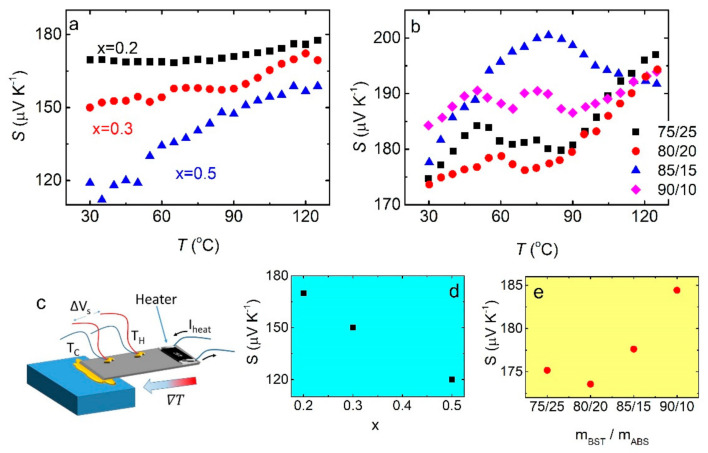
(**a**) $S\;\left(T\right)$ curves for BST/ABS blends mBSTmABS= 8020 for various BST compositions. The experimental configuration for Seebeck coefficient measurements is shown in the inset. (**b**) Room temperature S values, with respect to the Bi doping. (**c**) Schematic demonstration of the experimental set-up, for Seebeck coefficient measurements. (**d**) $S\;\left(T\right)$ curves for BST/ABS blends (BST x = 0.4) for several mass ratios. (**e**) Room temperature Seebeck coefficient vs. BST (x = 0.4)/ABS mass ratio, as extracted from panel **b**.

Figure 6a shows the temperature dependence of the Seebeck coefficient, for BST/ABS bends, in various BST dopings mBSTmABS= 8020. In all cases, S increases with temperature. Moreover, S decreases with Bi doping, consistent with previous reports (Figure 6d). Absolute values of the Seebeck coefficient are slightly lower than those for bulk materials [17] as well as other polymer/BST composites [29,34]. Therefore, in contrast to the increased resistivity, the Seebeck coefficient seems to be hardly affected neither by the fine BST grain size nor by the high porosity. Notably, the charge carrier conduction is severely affected by the presence of grain boundaries resulting in enhanced resistivity, as well as an increased Seebeck coefficient, with decreasing grain size [36,37]. Moreover, porosity also enhances the Seebeck coefficient [35], in contrast to the observed slightly reduced S values. Further investigation is required to clarify such a contradiction; however, it is beyond the scope of the current study. In addition, the Seebeck coefficient vs. temperature for mixtures with several BST (x = 0.4)/ABS mass ratio is shown in Figure 6b. In general, S tends to increase with increasing T. Moreover, S increases with an increasing BST/ABS mass ratio (Figure 6e).

**Figure 7:**
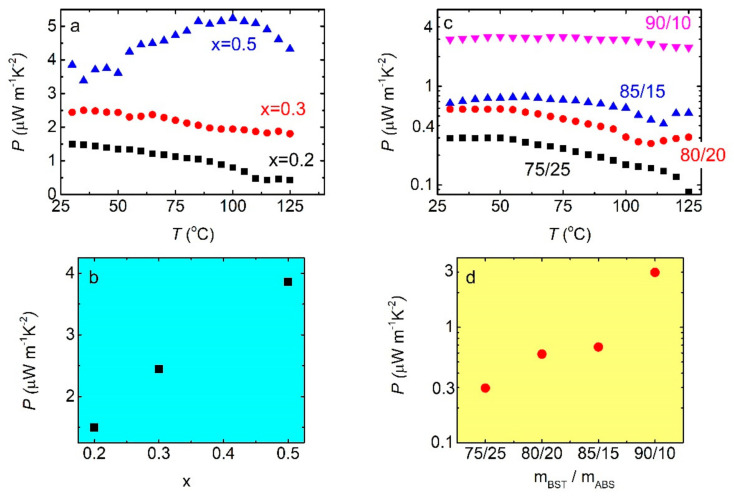
(**a**) Thermoelectric power factor vs. temperature, for BST/ABS blends mBSTmABS= 8020 and for different BST compositions. (**b**) Room temperature P values, with respect to the Bi doping. (**c**) $P\left(T\right)$ curves for BST/ABS blends (BST x = 0.4), for several mass ratios. (**d**) Room temperature P vs. mass ratio, as extracted from panel **c**.

Figure 7a depicts the temperature dependence of the thermoelectric power factor for BST/ABS blends, with different Bi doping levels. P tends to decrease with *T*, for x = 0.2 and x = 0.3 samples, while it slightly increases for x = 0.5 sample. The latter sample also exhibits the highest P values of the three samples in the whole temperature range. Decrement of P with respect to the temperature has been reported for BST powders [38,39]; therefore, such behavior is an intrinsic property of the BST material. P increases with increasing Bi doping, as shown in Figure 7b. Such behavior agrees with others reported in the literature for pure BST materials [38,40]. On the other hand, there are reports where P shows a local maximum for x = 0.3–0.5, and they are in contrast with our findings [23,39]. In addition, absolute P values are comparable to those reported for other polymer-thermoelectric material composites produced through complicated processes [11,12,13,14]. Thus, BST/ABS foils produced by mechanical mixing and hot pressing are an effective method for the production of polymer-thermoelectric materials in the form of thin foils. However, herein reported P values are 3–4 orders of magnitude lower than those reported for bulk BST materials [17,38,39,40]. Furthermore, as shown in Figure 7c, P decreases with increasing T for all BST (x = 0.4)/ABS, regardless the mass ratio. Even more, P increases with increasing BST loading into the blending (Figure 7d). Such behavior resembles the behavior reported for a PLA/BST (x = 0.5) composite [34].

**Figure 8:**
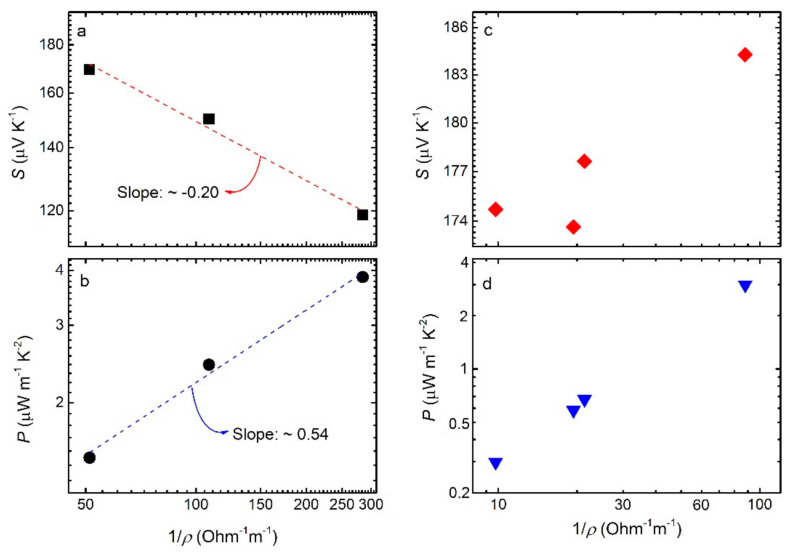
(**a**) Seebeck coefficient vs. electrical conductivity, for BST/ABS blends mBSTmABS= 8020 and for different BST compositions. Red dash line corresponds to S ∝ σ−14 fit. (**b**) Thermoelectric power factor vs. electrical conductivity. Blue dash line denotes the P∝ σ12 fit. (**c**) Seebeck coefficient vs. electrical conductivity and (**d**) Thermoelectric power factor vs. electrical conductivity curves for BST/ABS blends (BST x = 0.4), for several mass ratios.

The Seebeck coefficient as a function of electrical conductivity σ (electrical conductivity equals to inverse resistivity i.e., σ=1/ρ, for BST/ABS blends mBSTmABS= 8020 and various Bi compositions, is shown in Figure 8a. S decreases with increasing *σ.* Linear fit of log(*S*) against $log\left(\sigma \right)$ gives a slope of $-\left(0.20\pm 0.03\right)$. Hence, data seem to roughly follow the empirical relation $S\;\propto \;\sigma^{-1/4}$ [41]. Furthermore, power factor *P* is plotted against *σ* in Figure 8b. Linear fit of log(*P*) against $log\left(\sigma \right)$ gives a slope of $-\left(0.54\pm 0.05\right)$, which corroborates with the empical relation $P\;\propto \;\sigma^{-1/2}$. Therefore, the behavior of the Seebeck coefficient, and consequently the power factor, in respect to conductivity follows the similar general trend as several other semiconducting polymers and polymer nanocomposites [42]. In our case, the semiconducting character of the blend most likely comes from the BST component, which is considered a semimetal, rather than from ABS, which is a pure insulator. Figure 8c shows the Seebeck coefficient as a function of electrical conductivity for BST/ABS blends (BST x = 0.4) with different mass ratios. Here, *S* increases with increasing *σ*, and therefore it cannot follow the $S\;\propto \;\sigma^{-1/4}$ tendency. As a result, the *P* vs. *σ* plot (Figure 8d) cannot be described by the relation $P\;\propto \;\sigma^{-1/2}$. Most likely, as the ABS loading decreases BST/ABS blends tend to behave as pure BST materials rather than nanocomposites, thus the empirical relations cannot be applied.

## 4. Summary and Conclusions

In the current study, mixtures of the polymer ABS and Bi_x_Sb_2-x_Te_3_ thermoelectric material were produced employing a simple mechanical mixing procedure, without using any liquid solvent. Several blends were produced with different BST/ABS mass ratios. Furthermore, BST/ABS mixtures were produced, where the mass ratio was kept constant, while BST with different Bi composition was used. These blends were then hot-pressed so that thin sheets were fabricated.

These foils were characterized through SEM experiments where softly segregated clusters of BST grains were observed. Moreover, melted ABS seems to surround BST grains, indicating that mechanical mixing of BST and ABS powders results in qualitative blended foils. The crystalline structure of BST/ABS foils was confirmed by XRD experiments. The obtained XRD patterns show diffraction peaks that are attributed to the BST crystal structure, suggesting that the hot-pressing procedure does not affect the BST crystal structure. ABS does not exhibit any crystallinity, and thus no corresponding XRD peaks can be found in the XRD pattern. The atomic structure of the BST/ABS blends was explored using Raman spectroscopy. Peaks corresponding to BST vibrational modes were identified in the obtained Raman spectra, indicating the pure BST phase exists in the blends.

Transport properties of all the formatted foils have been measured in the temperature range going from room temperature up to 130 °C. Considering their resistivity, all samples show metallic behavior. Moreover, for the constant BST/ABS mass ratio 80/20, resistivity decreases with increasing Bi doping. Both these findings are characteristic of pure BST material, as reported in the literature. In addition, resistivity is suppressed by increasing the BST load into the blending. Absolute resistivity values are superior to others reported for polymer/BST composites; nevertheless, they are much higher than resistivity values of pure BST materials. Such a great difference is most likely attributed to the fine grain nature, along with the high porosity observed in the investigated foils.

Regarding the Seebeck coefficient, all samples show positive S values, suggesting the p-type charge character of the BST/ABS blends. For a constant BST/ABS mass ratio, S increases with T, while absolute S values are slightly lower than those obtained for pure BST materials. Furthermore, S decreases with increasing Bi doping, such as in bulk BST. The fact that thermoelectric power is slightly affected by the presence of ABS is advantageous, since it demonstrates that the produced polymer-based composite sustains the thermoelectric properties of the TE inclusion, as it was initially desired. In addition, S increases with increasing BST loading into the composites. Maintaining S values high enough upon BST load reduction appears to be a challenge.

The resulting thermoelectric power factor indicates that BST/ABS mixtures exhibit thermoelectric performance comparable to other polymer-based thermoelectric composites. Therefore, mechanical mixing is an effective alternative method for the production of composites dedicated for thermoelectric applications. On the other hand, the obtained thermoelectric power factors are considerably lower than those obtained for bulk BST materials. Such a great difference is mainly attributed to the dramatically increased resistivity exhibited in the BST/ABS composites, in comparison to bulk BST. Therefore, further investigation is required to explore possible ways for the resistivity decrement, such as the decrement of the foil porosity, the use of large grain BST powders (to reduce grain boundary effects), as well as the incorporation of a third (conductive) material into the composite.

In conclusion, mechanically mixed and hot-pressed BST/ABS composites can be alternatively promising candidates towards the construction of flexible and arbitrary-shaped thermoelectric devices.

## Data Availability

All data reported here can be made available on request.
